# Determining Abuse Deterrence Performance of Poly (ethylene oxide) Using a Factorial Design

**DOI:** 10.15171/apb.2018.058

**Published:** 2018-08-29

**Authors:** Yogesh Joshi, Srinath Muppalaneni, Alborz Omidian, David Jude Mastropietro, Hamid Omidian

**Affiliations:** ^1^Unipharma LLC., Tamarac, Florida, USA.; ^2^Sancilio Pharmaceuticals Company, Inc., Riviera Beach, Florida, USA.; ^3^The University of Chicago, Chicago, IL, USA.; ^4^Department of Pharmaceutical Sciences, College of Pharmacy, Nova Southeastern University, Fort Lauderdale, Florida, USA.

**Keywords:** Abuse deterrent formulation, Injecting drug use, Crush resistance, Opioid abuse, Poly(ethylene oxide)

## Abstract

***Purpose:*** The purpose of this study was to determine the effects of thermal processing and antioxidant formulation variables on the abuse deterrence performance of a high molecular weight poly(ethylene oxide) (PEO) polymer.

***Methods:*** A 2^4^ factorial design with one categorical factor (antioxidant type) and three continuous factors (curing time, curing temperature, % antioxidant) was used. Abuse deterrence performance was evaluated using solution viscosity, surface melting temperature, and mechanical strength. Thermal degradation of PEO powders before compaction was also studied using DSC, FTIR spectroscopy, and viscosity analysis.

***Results:*** Our results showed that curing temperature and type of antioxidant can significantly affect the deterrence performance of PEO. The main effect plot for viscosity shows the most prominent factors affecting viscosity are curing temperature and type of antioxidant. However, curvature in the linear model obtained was not sufficient to completely describe the behavior. For surface melting temperature, butylated hydroxytoluene was associated with higher surface melting temperatures compared to ascorbic acid. Additionally, higher percent of antioxidant resulted in higher melting temperature. Particle size distribution to indicate mechanical strength showed no significant effects of tested factors. This suggests that comminution method has more prominent effect on tablet fragment size than the formulation and processing factors studied.

***Conclusion:*** While heat confers the mechanical strength to the polymer, it can diminish its physical stability and solution state viscosity. The experimental studies showed that prolonged exposure to high temperatures, even in the presence of antioxidants, can severely hamper polymer deterrence performance in both solid and solution states.

## Introduction

The increasing use of prescription drugs in the United States suggests their major importance to clinical practice.^1^ Along with their increased use, there has also been a rise in abuse of certain classes of medications, among which the prescription opioid pain relievers are perhaps the most widely-abused. For example, it has been reported that approximately 97.5 million people aged 12 or older were past year users of prescription pain relievers in 2015.^2^ This represents more than one third (36.4 percent) of the United States population aged 12 and older. Additionally, misuse of prescription opioids as well as other classes has led to increased emergency room visits, overdose deaths, and treatment admissions for drug use disorders and addiction.^3^ Furthermore, the rise in prescription drug abuse has also been associated with increased economic burden, particularly related to criminal and legal costs.^4^ Therefore, methods and technologies aimed at decreasing such abuse are being actively researched and implemented.

One such technology uses unique dosage forms that are meant to decrease the abuse potential of certain medications such as opioids. Products equipped with these abuse deterrent features are commonly called abuse deterrent formulations (ADFs). It is believed that these formulations have the potential to decrease abuse without limiting access of opioid prescriptions to legitimate patients.^5^ In general, ADFs lower the abuse desirability of a medication by preventing physical (e.g., crushing, chewing) and chemical (e.g., drug extraction) tampering, prevent drug metabolism or binding, or incorporate aversive materials (e.g., bittering agents, mucous membrane irritants) into the product.^6^ Of these, those that physically and chemically hinder tampering have become most predominant in the market. Furthermore, within this category of commercialized ADFs, we see an extensive use of high molecular weight poly(ethylene oxide) (PEO).^7^ Several formulations with approved ADF labeling use technologies that employ PEO as a primary means of providing abuse deterrence. These include opioid formulations such as Oxycontin, Targiniq ER, Hysingla ER, and Arymo ER.^8^

The popular use of PEO as a pharmaceutical excipient is based on several factors. PEO is associated with a high LD_50_ value, and therefore generally considered non-toxic for oral use.^9,10^ Its non-ionic structure also provides a low tendency for drug interactions when used as a matrix for drug delivery. Additionally, it can easily be formulated into solid oral dosage forms (such as tablets) due to its good compressibility and lubricity characteristics. The low melting temperature of PEO (Tm, 65 – 70^o^C) allows extrusion, molding, and casting of solid dosage forms containing large amounts of the polymers.^11^ PEO has several other desirable properties that can explain its prevalent use in ADFs. For example, PEO is a water-soluble polymer that is available in low (as low as 100,000 Da) to very high (7,000,000 Da) molecular weights. PEO hydrates rapidly in aqueous solvents and continues to swell to a large extent to form a thick gel-like solution,^12^ that is believed to deter abuse by injection. Therefore, higher molecular weight PEOs are often used in ADFs to produce enhanced viscosities needed for abuse deterrence. PEO also shows pH-independent hydration, meaning it will provide viscosity in many different types of solvents that abusers may use for extraction processes. However, high molecular weight PEO solutions have been shown to exhibit some shear thinning behavior at high shear rates.^13^ Since high shear stresses can occur when a PEO solution is aspirated into a syringe, abusers may still find it easy to abuse by injection despite a high viscous appearance. Furthermore, the ease of injecting a PEO solution is also evident by other studies showing parenteral drug delivery applications and injectable in-situ gel-forming systems based on PEO.^14,15^ It is also known that the rheology of PEO solutions is largely influenced by hydrogen bonding in the solvent, as well as between the solvent and polymer.^16^ Consequently, different solvents used during abuse may have variations in polarity or electrolyte concentrations that may significantly affect the rheological properties of aqueous PEO solutions. Even more, temperature can also influence the viscosity of PEO solutions. The effects of these solution variables are discussed further in the results section of this paper.

In terms of abuse deterrence, PEO has also been used to increase the mechanical strength of tablet dosage forms through thermal processing. For example, PEO-based matrix tablets can be exposed to a heat curing process after compression or manufactured directly using hot melt extrusion. Here, curing is a process where the temperature of the compressed tablets is brought up close to the melting temperature of the excipient polymer(s). Upon cooling, the polymer solidifies and imparts a plastic-like effect to the tablets with improved mechanical strength. Thermally processed PEO tablets are highly resistant to crushing, chewing, and grinding by common household instruments (e.g., blenders, coffee grinders, and hammer). The effectiveness of this thermal curing process is perhaps best highlighted by the prescription opioid OxyContin® ER. The reformulated version of this product currently has the widest market coverage, and its post-marketing data suggests a significant intervention to abuse.^17-19^

PEO used in hot melt extrusion is generally supplied with some required amounts of antioxidant (e.g., butylated hydroxytoluene) due to its susceptibility to thermo-oxidative degradation.^20,21^ Temperature controlled manufacturing processes can be used to mitigate this process. However, in the realm of abuse, there will be no limit as to how long or at what high temperatures abusers may tamper with the medication. Abusers have been known to use microwares, stoves, and other heating devices to degrade the PEO polymer structure, and hence defeat its abuse deterrence properties.

Since most current ADFs are PEO-based and thermally manufactured, we aimed to investigate major factors during this process that may have the most significant effect on the final abuse deterrent properties of such a product. The objective of this study was therefore to analyze the effects of certain formulation and processing factors on the integrity of PEO-based compositions in terms of their abuse deterrence performance. This was done using a 2-level full factorial design with four chosen factors related to a curing process: 1) curing temperature, 2) curing time, 3) type of antioxidant, and 4) antioxidant percent. We measured abuse deterrence in terms of the effects of these parameters on solution viscosity (extraction potential), mechanical strength (crush resistance), and thermal behavior/stability (resistance to drug volatilization) of tablet compacts.

## Materials and Methods

PEO (Sentry™ Polyox^™^ WSR-303) with a Mol. Wt. of 7,000,000 Da was obtained from Dow Chemical Inc. (Midland, MI, USA). Ascorbic acid and butylated hydroxytoluene were obtained from Amresco LLC. (Solon, OH, USA) and Sigma-Aldrich Inc. (St. Luis, MO, USA), respectively. Ultrapure water (≈ 18 MΩcm) from in-house Milli–Q® system (Bedford, MA, USA) was used for all aqueous solutions. All other chemicals were of analytical grade, and used as received.

### Factorial design

In this study, a 2-level full factorial design with 4 factors was chosen. Three of the factors were continuous (*i.e.,* curing time, curing temperature, % antioxidant), and one was categorical (*i.e.,* antioxidant type). Therefore, a 2^4^ full factorial experimental design where each factor was investigated at two levels (low and high) was generated. Each factor level and type were chosen based on preliminary experiments. Additionally, center points (one for each categorical factor) were also incorporated into the design to detect possible curvature in the fitted data. Three response variables (*i.e.,* viscosity, surface melting temperature, % particles >850 µm) were chosen to be measured for each run as a measure of abuse deterrence performance. Stepwise regression analysis of the factorial design was performed using Minitab 18.1 software to determine the main effects and possible interactions between factors. The stepwise regression procedure we selected systematically adds to the model the most significant variables and removes the least significant variables. Using no hierarchy restrictions, this process stops when all variables that are not in the model have *p*-values that are greater than that specified (i.e., α = 0.05).

### Preparation of Tablet Compacts

To eliminate the effects of several processing excipients, direct compression tablets were made of only PEO with or without an antioxidant to a total weight of 200 mg. Based on the amounts dictated in the experimental design, a physical mixture of either 0%, 1%, or 2% antioxidant (ascorbic acid (AA) or butylated hydroxytoluene (BHT) was combined with PEO. The powder mixtures were then compressed into tablets using a single station Carver press (Carver Inc., IN, USA) with a ½ inch diameter die and standard concave tooling at a compression force of 2000 lb. We refer to these compressed powder mixtures as tablet compacts in this paper. It should be noted that the PEO used in these experiments already contained 100-500 ppm BHT as claimed by the manufacturer.^22^

### Thermal Curing

Each tablet compact was subjected to curing at various temperatures and time based on the factorial design. The curing step was performed inside an air recirculated oven with each tablet compact returned to room temperature before any experiments were conducted.

### Evaluation and Characterization of Tablet Compacts

#### Viscosity Measurements

Tablet compacts were broken into fragments and dissolved in an appropriate amount of water to obtain a 1% w/v solution. Viscosities of the resultant solutions were measured using a cone and plate rheometer (Brookfield DV-III Ultra) at a shear rate of 300 sec^-1^ for 40 sec. We used viscosity values as an indicator of abuse deterrence via extraction for subsequent injection. The greater the viscosity, the greater resistance to syringeability was presumed.

#### Surface Melting Temperature 

After curing, surface shavings from the tablet compacts were obtained for thermal analysis. Thermograms of the shavings were obtained using differential scanning calorimeter (DSC 4000, Perkin Elmer). Briefly, 10 mg of sample was weighed in a flat-bottomed aluminum pan and placed in the furnace. The sample was subjected to a gradual temperature increment and decrement cycle at the rate of 10^o^C/min from 25 to 250^o^C under a nitrogen gas purge (20 mL/min) to maintain an inert environment. The melting point was used as an indicator of solid state thermal stability and likelihood of drug volatilization. High thermal melting temperature was assumed to be associated with greater prevention of crushing and degradation of the product in the solid state.

#### Mechanical Strength 

The resistance of cured tablet compacts to particle size reduction (mechanical strength) was determined using a high shear grinder (MicroMill® II, SP Scienceware Inc., NJ, USA). Each sample was placed alone in the mill and subjected to a steel blade revolving at full speed (≈10,000 rpm) for 60 seconds. Immediately following grinding, the contents were sieved to determine particle size distribution via sieve analysis. We considered the percent of fragments greater than 850 µm as an indication of crush resistance by commonly available household comminution practices.^23^ We therefore used “% of fragments >850 µm” throughout the paper as an indicator of tablet compact mechanical strength. The higher the percentage of larger particles (>850 µm), the greater is the resistance to crushing, chewing, and grinding.

#### PEO Powder Thermal Degradation Properties

To further characterize the thermal properties of PEO, the following studies were performed on PEO powder.

#### FTIR Analysis

Infrared spectroscopy (FTIR Spectrum 100, Perkin Elmer) was used to observe any structural changes that may be occurring during the curing step. Spectra were obtained between wavelengths 650 and 4000 cm^-1^ on PEO powders that were exposed to different hot-air temperatures (80, 110, 150, and 180^o^C) for 1 hour.

#### DSC Analysis

DSC experiments were conducted using PEO powder to observe thermal behavior and degradation during heating in the presence of nitrogen and air using the same method as described previously.

#### Viscosity Analysis

Viscosities of 2% w/v aqueous solutions of non-heated and heat-treated PEO powder samples using the cone and plate rheometer procedure as previously described were obtained to observe any loss of viscosity caused by thermal degradation. Furthermore, the effect of solution temperature on the viscosity of PEO solutions was also performed using 0.5, 1, 2, 2.5, and 5% w/v PEO aqueous solutions at solution temperatures of 25, 50, and 90^o^C.

## Results and Discussion

The influence of two formulation factors (antioxidant type and concentration) and two processing factors (curing temperature and curing time) on the final abuse deterrence performance of PEO-based tablets was evaluated using ANOVA and factorial plot graphs. The graphs included main effect, interaction, cube, and Pareto. Results for all runs are shown in Table 1.

**Table 1:** Full factorial design matrix and response parameter results

Run Order	Anti-oxidant Type	Anti- oxidant (%)	Curing Temp (oC)	Curing Time (min)	Viscosity (cP)	Surface melting temp (oC)	% of tablet fragments >850 μm
12	AA	2	25	180	2.4	76.89	64.25
2	AA	0	25	30	63	78.67	89.76
11	BHT	2	25	180	0	76.36	49.45
10	AA	0	25	180	2.18	74.45	58.28
1	BHT	0	25	30	58.64	80.89	83.77
4	AA	2	25	30	33.13	78.29	59.3
3	BHT	2	25	30	63	77.74	55.44
9	BHT	0	25	180	0.43	76.85	62.69
18	AA	1	87.5	105	8.5	69.82	34.67
17	BHT	1	87.5	105	2.83	71.55	68.84
7	BHT	2	150	30	2.18	71.22	62.88
8	AA	2	150	30	0.43	70.48	60.51
5	BHT	0	150	30	28.99	72.79	81.63
16	AA	2	150	180	83.71	76.65	86.36
6	AA	0	150	30	106.6	68.4	92.57
14	AA	0	150	180	33.13	62.42	77.49
15	BHT	2	150	180	2.83	74.41	86.15
13	BHT	0	150	180	54.5	65.58	76.24

### Analysis of Factorial Design 

For our 2^4^ full factorial experiment, it would be possible for each response variable to have a model fit containing a mean term, four main effect terms, six two-factor interaction n terms, four three-factor interaction terms, and a four-factor interaction term (16 parameters). With this high number of terms, and the difficulty of interpreting higher order interaction terms existing at significant levels, a stepwise approach was chosen. The stepwise approach starts with a simple model having only the mean, and then adds or removes terms in a “stepwise” manner. Variables were deleted from the model if they had *p*-values greater than 0.05 or kept if their *p*-values was less than or equal to 0.05. This was done to keep only those factors in the model, which had the most significant main effects and interactions.

**Figure 1:**
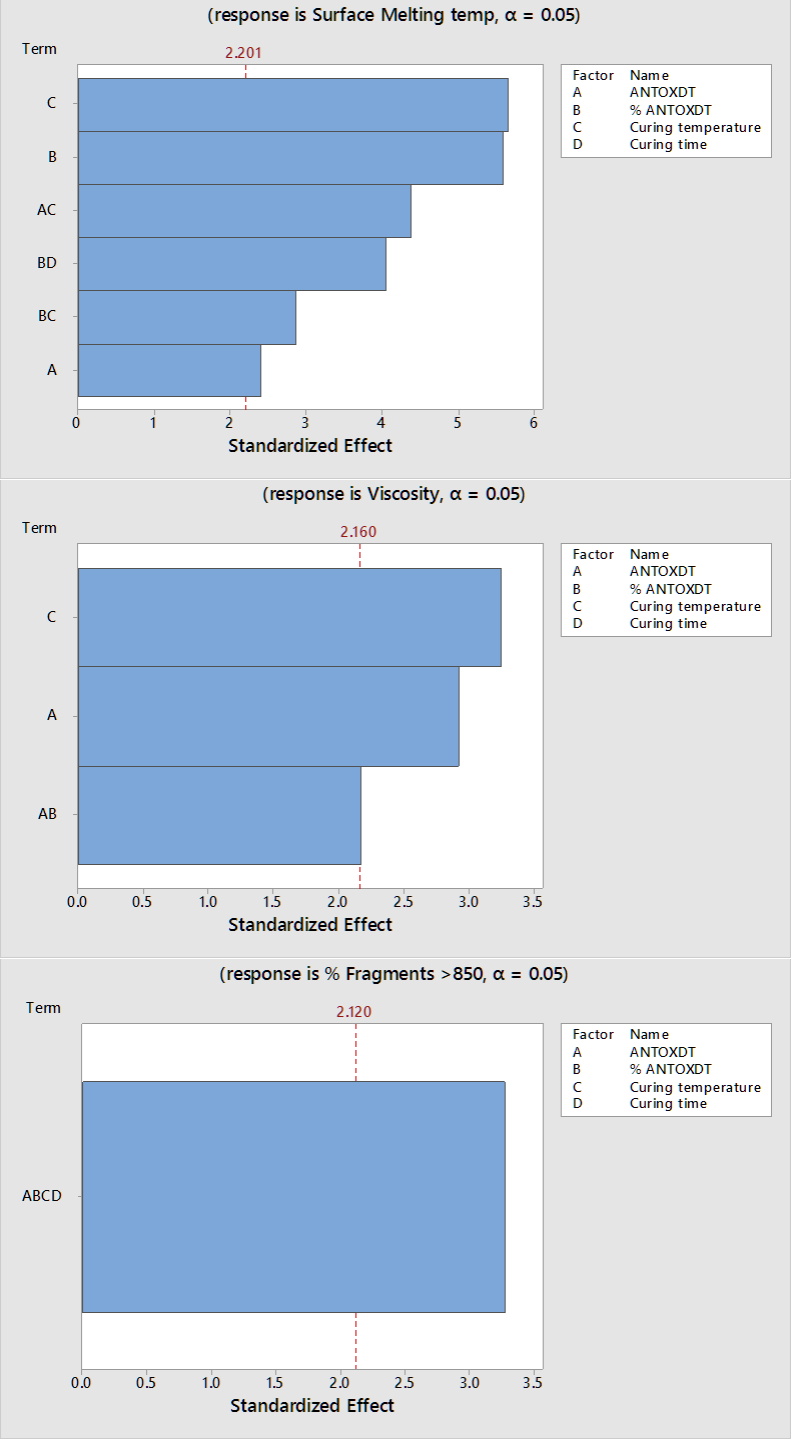
Pareto Charts of standardized effects

ANOVA results for each model showed not all factors and interaction terms were significant. Pareto charts showing only the significant terms and their relative importance for each model can be seen in Figure 1. The length of the horizontal lines in the charts are representative of the factors difference from zero. The vertical reference line indicates the critical value (*p* = 0.05), where a bar extending to the right indicates its significance.

The top graph in Figure 1 shows curing temperature and percent antioxidant were the most significant main effect factors influencing surface melting temperatures along with antioxidant type. Additionally, three 2-way interaction effects were also represented. For example, the surface melting temperature is also influenced by the relationship (interaction) between the type of antioxidant chosen and curing temperature, percent antioxidant and curing time, and percent antioxidant and curing temperature. These types of interactions would be expected since surface melting temperature might be affected, for example, not only by the percent of antioxidant but also the time or temperature of the curing process.

Similar to above, the middle graph in Figure 1 shows the response variables for viscosity. In this case, curing temperature and type of antioxidant were the only main effect variables shown to be significant. Furthermore, the type of antioxidant and percent antioxidant were the only 2-way interactions most influencing viscosity. In this case, the more ascorbic acid in the formula the lower the resultant viscosity compared to BHT. In the final graph of Figure 1, we see only a 4-way interaction for the model defining % of fragments >850 µm, which is challenging to interpret meaningfully.

Polynomial equations represented by the significant terms and interacts for all models are shown in Table 2. These functions represent how the independent variables and their interactions influence the response tested. The positive or negative value of each coefficient, along with its relative value, dictate how much of an increase or decrease will occur in the parameter being measured. The R^2^ values representing the variations explained by the models were well fit for surface melting (91.13%) and less for viscosity (70.41%). The closeness of the values between R^2^ and predicted R^2^ for surface melting temperature shows how well the model predicts the response. However, the larger difference between these variables for viscosity indicates the likelihood the model is only predicting the observed data and not that of the true population. The ability of the model to fit the data from fragments >850 µm was very low (40.15%) and not relevant.

Additionally, the residual errors were partitioned into pure error, curvature, and/or lack of fit. The use of center points in the design allowed us to determine curvature in the fitted data. Curvature occurs when the mean response is greater or less than the corner points. While the lack of fit sum of squares, encompasses the effect of omitted interaction terms. We can see that for viscosity, curvature is suspected due to the low *p*-value and may explain why the model is not very predicative.

**Table 2:** Polynomial regression equations for response variables

**Response**	**Equation**	**Model Summary**			
		**S**	**R-sq**	**R-sq** **(adj)**	**R-sq (pred)**
Surface Melting Temp	= 73.539 - 1.028 ANTOXDT + 2.544 % ANTOXDT - 2.569 Curing temp - 1.994 ANTOXDT*Curing temp + 1.306 % ANTOXDT*Curing temp + 1.844 % ANTOXDT*Curing time	1.817	91.13%	86.30%	75.03%
Viscosity	= 25.68 - 14.40 ANTOXDT - 16.99 Curing temp- 11.35 ANTOXDT*% ANTOXDT + 42.1 Ct Pt	20.940	70.41%	61.31%	27.85%
% Fragments >850	=69.46- 10.28 ANTOXDT* % ANTOXDT*Curing temp*Curing time	12.553	40.15%	36.41%	23.85%

### Surface melting Temperature

**Figure 2:**
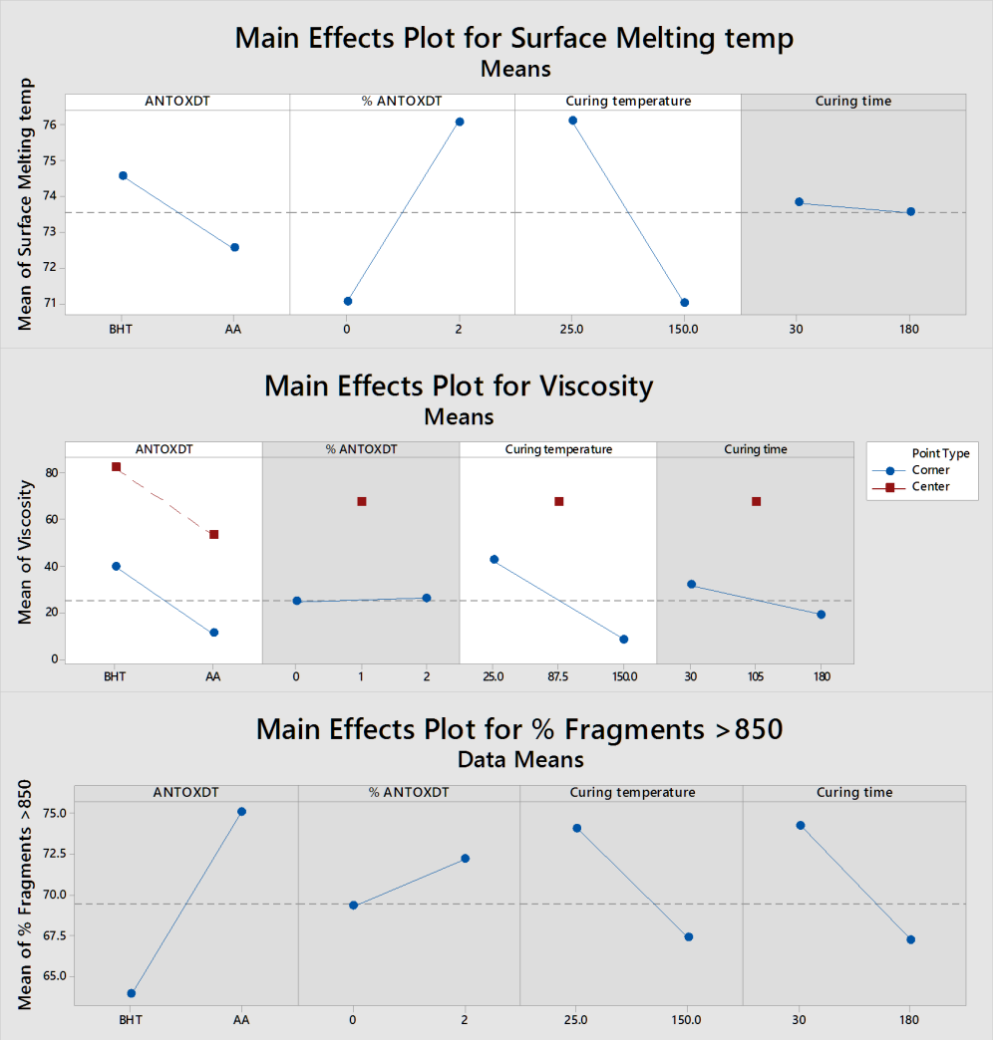
Main effect plots for response variables (a gray background represents a term not in the model)

The temperature at which scrapings from the surface of the tablet compacts in the experimental design began to melt was used to assess thermal stability and as a way of measuring resistance to drug volatilization. For volatilization, we are assuming a soften or melted polymer would release drug easier when heated under abuse conditions used for this type of abuse. A plot of main factor effects for surface melting are shown in Figure 2. Main effect plots help in understanding how the response is affected by varying only one factor while keeping others constant. Main effect is indicated when average response changes across the levels of a factor. The main effect plot is created by plotting response against all levels of factors. If a line connecting the mean responses at factor levels is horizontal (parallel to X axis), it shows absence of main effect. However, if the line is not horizontal, and greater the slope of the line is, greater is the magnitude of the main effect.^24,25^

The significant main factors were antioxidant type, antioxidant percentage, and curing temperature. From Figure 2, we see that BHT is associated with higher surface melting temperatures compared to AA. Additionally, a higher percent of antioxidant will rise to a higher melting temperature. Since the antioxidant is used to prevent polymer degradation, a higher percent would likely result in a higher temperature of melting as the polymer molecular weight is better preserved. The beneficial effects of BHT on PEO stability over AA indicates that the primary source of oxidation reaction occurring on PEO at higher temperature are free radicals rather than oxygen. Curing temperature was also shown to be significant, meaning if PEO has been subjected to a thermal process, its melting temperature on re-heating (during abuse) would be less. This might suggest that ADFs may not need to be thermally treated to prevent melting, extraction, and drug volatilization. However, mechanical strength would likely be lower in such products. The Pareto chart for surface melting shows that 3 main effect terms (antioxidant type, antioxidant percent, and curing temperature) were significant, with curing temperature and percent antioxidant have greatest effects. However, the regression equation for surface melting shows that the impact of these to be minimal based on the low values of the coefficients. This may explain why the R^2^ values are high. The highest interaction coefficient between antioxidant type and curing temperature (-1.994) may be explained based on the stability of the antioxidants at higher temperatures. The positive coefficients for the interaction of curing time and temperature with percent of antioxidant are also likely to be based on the lower degradation when lower temperatures and time are used along with the higher percent of antioxidant. When a response at one factor level depends on the levels of other factors, it shows an indication of an interaction. Interactions are critically important as they can reinforce or cancel out the main effects of factors. Thus, main effect cannot be interpreted without considering interaction effects.

The changes PEO experiences during a thermal event were also visually noticeable when exposed to heat curing having no additional antioxidant added. At high temperatures, the white polymer began to turn into a pale yellow starting at 150^o^C, indicating oxidative degradation. The pale yellow turned noticeably darker for samples exposed to 180°. Additionally, as the polymer began to soften and melt, the cohesive forces between powder particles were also increased and became more rigid on cooling. Furthermore, DSC analysis of PEO samples which were once heated (pre-heat treated) at different temperatures, showed gradual shifts in melting point peaks towards lower temperatures. The peak melting temperatures for these samples pretreated at 80°, 110°, 150°, and 180°C were 72.48°, 71.07°, 65.20°, and 54.65°C, respectively. Melting peak onset as well as heat of fusion were also decreased with an increase in temperature of the treatment. Therefore, it may be beneficial to manufacture an ADF composed of PEO at the lower temperatures to maintain greater resistance to thermal methods of tampering.

**Figure 3:**
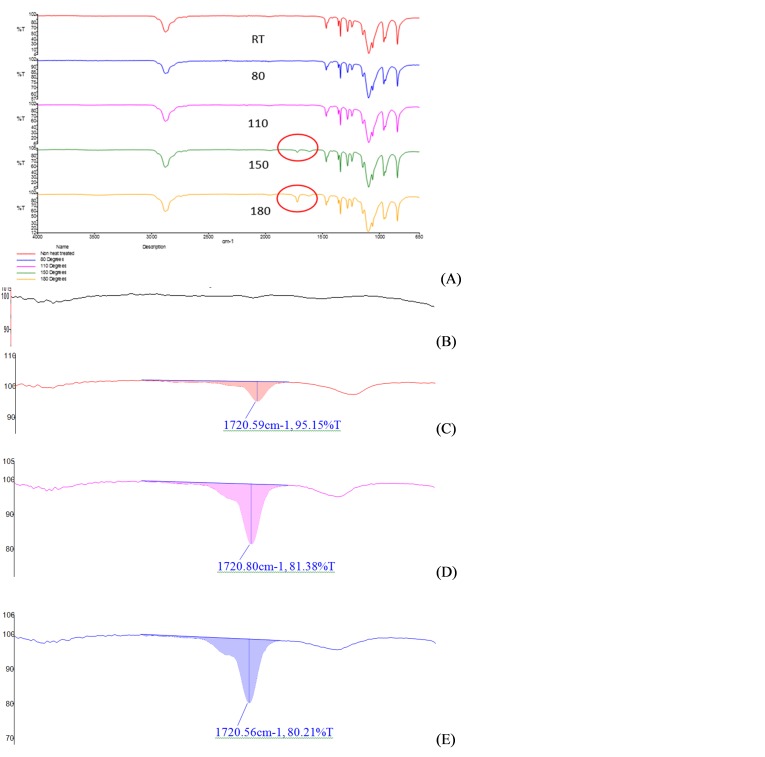
FTIR spectra of heat treated PEO (Polyox^TM^, WSR coagulant) in solid state indicating oxidative degradation at higher temperatures (A), Magnification of degradation peak of PEO sample heat treated at (B) 110^o^C (B) 150^o^C, (C) 180^o^C for 1 hr and (D) 150^o^C for 3hrs at 1720 cm^-1^

Further analysis via FTIR spectra of heat treated PEO samples showed appearance of a degradation peak at a wavelength of 1720 cm^-1^ for temperatures ≥ 150^o^C (Figure 3 (A)). This peak was absent in the spectrum at 110^o^C (Figure 3 (B)), whereas it shows prominent appearance at 150 and 180^o^C (Figures 3 (-C, -D, -E). Intensity of this peak was increased with increase in temperature as well as the duration of heat exposure (Figures 3 (-B, -C, -D). These observations provide significant evidence for PEO’s oxidative degradation at high temperatures and correlate with the DSC results. Our experimental design used 180^o^C as a high range curing temperature and may explain why the results may not fit well to our models.

### Viscosity 

The main effect plot for viscosity (Figure 2) shows the most prominent factors (greatest slope) affecting viscosity are curing temperature and type of antioxidant. This would be expected as higher temperatures would lead to greater polymer degradation and shorter end-to-end distance for the polymer chains. Therefore, processing at a higher curing temperature (or abuse at higher temperatures) would lead to lower viscosity of the resultant aqueous extract. Additionally, the presence of BHT in the formula seems to impart higher viscosity compared to AA with the concentration effect not being significant. However, the curing time had less effect on viscosity compared to the temperature used during curing. The impact of these factors determined using stepwise regression show only curing temperature and antioxidant type along with one interaction factor to be significant. The interaction effect may occur since having more antioxidant would allow for greater stability of the polymer at higher temperatures as well as contribute to higher viscosity. The main effect plot also shows that curvature may exist for this model as was evidenced by the ANOVA table showing significance for curvature (*p* = 0.019). The significant *p*-value for curvature strongly suggests that a linear model would not be most appropriate, and more points would be needed in the model completely describe the equation curvature. The regression equation provided by the software with the design space, includes a center point. This indicates that a particular curing temperature and time may exist where the antioxidant would have the most significant effect on viscosity of solutions made from the crushed tablet compacts.

**Figure 4:**
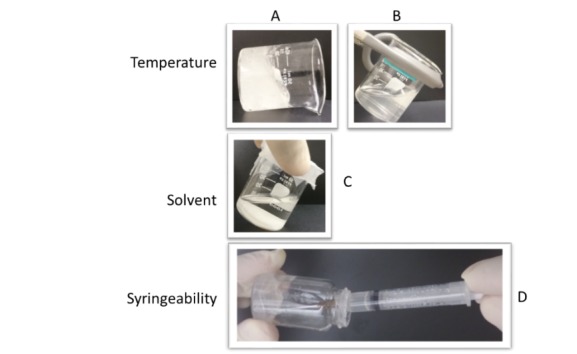
Susceptibility of a 2% w/v PEO solutions. Almost an 80% drop in viscosity was observed from at (A) ambient room temperature to (B) 95°C. (C) A complete precipitation was observed in ethanol. (D) The aqueous polymer (A) was easily syringeable at room temperature

Other factors not considered in the model, which can still affect the viscosity experienced by an abuser, are the type of solution being used and the temperature of the solution being extracted. The effects of these variables can be seen in Figure 4. Figure 4 (A) shows a very viscous 5% w/v PEO aqueous solution at ambient room temperature and when heated to 95^o^C, Figure 4 (B). This mimics a common practice by abusers in which a tablet extract solution is heated (e.g., lighter, candle) in order to enhance drug solubility prior to intravenous abuse. Figure 4 (C) shows the same concentration of PEO but made using pure ethanol instead of water as the extraction solvent. This time we see complete loss of viscosity as the polymer precipitates out of solution, as can be seen on the bottom of the glass vial. In Figure 4 (D), even though the 5% w/v aqueous solution of PEO at room temperature appears viscous, it could still be drawn into a syringe with an attached needle. This provides evidence that high solution viscosity may not be an effective deterrent for parenteral abuse.

**Figure 5:**
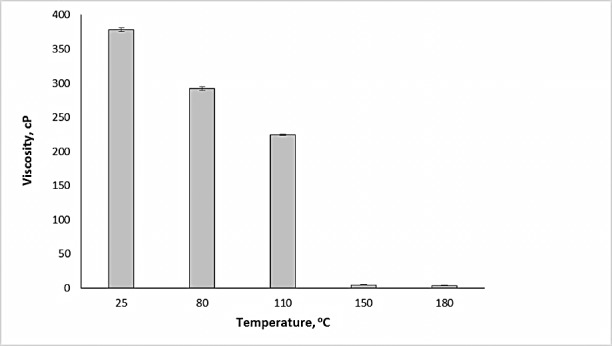
Drop in aqueous viscosity of PEO (Polyox^TM^, WSR, coagulant) treated at different temperatures (n=3)

Temperature dependent viscosity was also further influenced by the concentration of PEO in solution. The viscosity of PEO solutions (made from non-heat treated PEO) was found to increase non-linearly with increasing concentrations (i.e., 0.5, 1, 2, 3, and 5%). Furthermore, for the same concentration, the viscosity was found to be lower as the temperature was increased. When a 2% (w/v) PEO solution was made using a heat-treated sample, we saw a gradual decrease in solution viscosity up to a solution temperature of 110^o^C (Figure 5). As the temperature was further increased from 110 to 150^o^C, a sharp drop of about 97.9% in viscosity was observed, which continued upon heating further to 180^o^C.

Results of these studies show that PEO undergoes oxidative degradation especially at elevated temperatures. The oxidative degradation presumably resulted in breaking up of long linear chains in the polymer structure into small fragments, which resulted in the loss of polymer viscosity. These findings corroborate various stability and degradation studies previously conducted on PEO.^20,21,26-28^

### Mechanical Strength

Determining main factors that may influence mechanical strength (*i.e.,* % fragments >850µm) of a tablet compact showed no significant results of the factors we tested (Figure 2). Furthermore, the only interaction noted was a 4-way interaction that is hard to interpret at this high level (Figure 1). This resulted in a poor linear fit for the model at R^2^ of 40.15%. It may be likely we have not chosen factors that would have great impact on the mechanical strength after curing. Since thermal processing is used to impart mechanical strength to the polymer upon cooling, other mechanisms may also be involved. Furthermore, the high amount of PEO in the cured tablets made them very resistant to our particle size reduction efforts. We therefore believe the testing method used may have such a large variability that the results were not conclusive. From our prior experience developing methods to test crush resistance, we have noticed the standard error in such measurements has been wide despite controlling several variables. A separate study also suggested that method of comminution used had the most prominent effect on tablet fragment size when powerful electric instruments such as high shear grinders are used.^23^ Given this, we may conclude that grinding tablet compacts and further sieve analysis may not be the best determinant for such tamper resistant testing. The use of dynamic image analysis to assess the particle size of PEO based tablets crushed using a coffee grinder did show that the biggest factor affecting particle size was the curing temperature; higher curing temperatures and times resulted in more coarse particles.^29^ A similar study assessing the abuse deterrence of cured PEO based formulations found that as the percent of PEO increased, so did the crush resistance.^30^ However, the antioxidant type, antioxidant concentrations in the tablets, and curing temperatures were kept constant.

**Figure 6:**
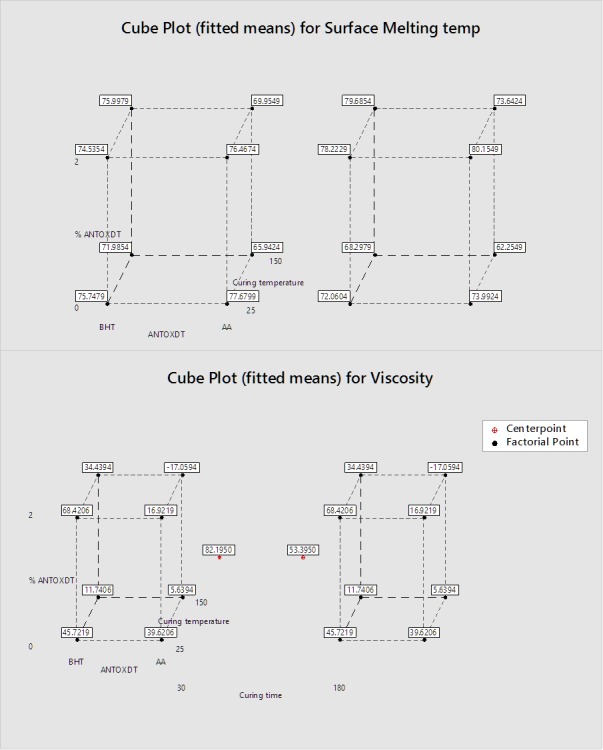
Cube plots for surface melting temperature (*top*) and viscosity (*bottom*)

In summary, this study used a full factorial design of experiments to determine the impact of 4 different processing factors on the final abuse deterrent properties of a product having PEO as a matrix base. We looked at major factors and interactions of these effects on the crush resistance (mechanical strength), solution viscosity of the extracting medium, and final melting temperature of the polymer-based tablet compacts. The crush resistance testing was not adequate to report meaningful results. However, the other results can most easily be shown in 3D cube plots (Figure 6), which represent average responses of melting temperature, and viscosity at critical points. Critical points in cube plots are the points where all factors have limiting values.^25,31,32^ Two cube plots were created for each response and represent two different curing times, 30 and 180 min. From the response cube plot of melting temperature, we can conclude that when curing is attempted at low temperatures, antioxidants do not impact the stability of the product. However, when the curing occurs at higher temperatures and for a longer period, the antioxidant BHT favors the surface melting at higher temperatures. The response cube plot for viscosity shows how the presence of BHT with or without heating also creates higher viscosity. Furthermore, the center points of this plot do not agree linearly as was previously discussed, making this model not ideal for full prediction. However, our results show that using BHT as an antioxidant and low curing temperature as well as shorter curing time would equip the solid dosage form with maximum resistance to abuse from tampering methods involving heat.

## Conclusion

Our data shows that high molecular weight PEO undergoes an oxidative degradation process at a rapid rate especially at high temperatures. This affects many aspects of polymer integrity particularly related to its deterrence performance. While heat confers the mechanical strength to the polymer, at the same time it diminishes its physical stability and solution state viscosity. The experimental studies showed that exposure to a high temperature for a long duration even in the presence of antioxidants can severely hamper polymer deterrence performance in solid and solution states.

## Acknowledgments

This article is based on research performed as part of a Ph.D. thesis written by Yogesh Joshi.

## Ethical Issues

Not applicable.

## Conflict of Interest

The authors declare that there is no conflict of interest regarding the publication of this article.
